# Evaluating MedDRA-to-ICD terminology mappings

**DOI:** 10.1186/s12911-023-02375-1

**Published:** 2024-02-07

**Authors:** Xinyuan Zhang, Yixue Feng, Fang Li, Jin Ding, Danyal Tahseen, Ezekiel Hinojosa, Yong Chen, Cui Tao

**Affiliations:** 1 McWilliam School of Biomedical Informatics, University of Texas Health Science Center at Houston, Houston, TX USA; 2 School of Engineering and Applied Science, University of Pennsylvania, Philadelphia, PA USA; 3 McGovern Medical School, University of Texas Health Science Center at Houston, Houston, TX USA; 4 The Perelman School of Medicine, University of Pennsylvania, Philadelphia, PA USA; 5 Department of Artificial Intelligence and Informatics, Mayo Clinic, Jacksonville, FL USA

**Keywords:** The medical dictionary for regulatory activities (MedDRA), International classification of diseases (ICD), Unified medical language system (UMLS), Observational medical outcomes partnership common data model (OMOP CDM), Terminology mapping

## Abstract

**Background:**

In this era of big data, data harmonization is an important step to ensure reproducible, scalable, and collaborative research. Thus, terminology mapping is a necessary step to harmonize heterogeneous data. Take the Medical Dictionary for Regulatory Activities (MedDRA) and International Classification of Diseases (ICD) for example, the mapping between them is essential for drug safety and pharmacovigilance research. Our main objective is to provide a quantitative and qualitative analysis of the mapping status between MedDRA and ICD.

We focus on evaluating the current mapping status between MedDRA and ICD through the Unified Medical Language System (UMLS) and Observational Medical Outcomes Partnership Common Data Model (OMOP CDM). We summarized the current mapping statistics and evaluated the quality of the current MedDRA-ICD mapping; for unmapped terms, we used our self-developed algorithm to rank the best possible mapping candidates for additional mapping coverage.

**Results:**

The identified MedDRA-ICD mapped pairs cover 27.23% of the overall MedDRA preferred terms (PT). The systematic quality analysis demonstrated that, among the mapped pairs provided by UMLS, only 51.44% are considered an exact match. For the 2400 sampled unmapped terms, 56 of the 2400 MedDRA Preferred Terms (PT) could have exact match terms from ICD.

**Conclusion:**

Some of the mapped pairs between MedDRA and ICD are not exact matches due to differences in granularity and focus. For 72% of the unmapped PT terms, the identified exact match pairs illustrate the possibility of identifying additional mapped pairs. Referring to its own mapping standard, some of the unmapped terms should qualify for the expansion of MedDRA to ICD mapping in UMLS.

## Introduction

Semantic interoperability is essential for big data analysis, whereby aligning different terminologies is the key to achieve interoperability among various data sources. In this era of big data, the same type of information can be stored in multiple data sources; however, not all of these sources adopt the same terminology, which makes it challenging to integrate or link these data sources for more comprehensive and powerful analyses. Linking heterogeneous data sources through terminology mapping is important for at least three types of tasks: (1) data integration to create large-scale datasets that link the data compiled in different terminologies [1]; (2) cross-validation to validate new signals detected from one data source with those of another data source [2]; and (3) research discovery to expand the scope of the existing research by including new information from different resources [3].

A common method for establishing reliable mappings between terminologies involves cross-referencing the target terminologies to a common vocabulary [4]. The Unified Medical Language System (UMLS) Metathesaurus and the Observational Medical Outcomes Partnership Common Data Model (OMOP CDM) are two common data models that are frequently used by the resesarchers to facilite terminology mapping [5–11]. The UMLS Metathesaurus, distributed by the U.S. National Library of Medicine (NLM), serves as a common dictionary that provides representations of biomedical concepts from nearly 200 different biomedical vocabularies [12] and is the largest thesaurus in the biomedical domain [13]. According to a survey conducted by the NLM, 49% of the UMLS users chose “facilitate mapping between terminologies” as their purpose [14]. Several studies have utilized UMLS as a tool to link different terminologies [10, 11, 15–19]. Different from UMLS, OMOP provides a data model to map different vocabularies to a common standard. The OMOP CDM was designed to conduct systematic analysis of disparate databases [20]. We used OMOP vocabulary under OMOP CDM for terminology mapping. Both OMOP vocabulary and the UMLS Metathesaurus can help to integrate vocabularies from different resources, whereas UMLS is more of a concept-based system in which all concepts are given a concept unique identifier (CUI).

There are three additional challenges to the use of these defined mappings in practice. (1) Different terminologies may focus on different subdomains and applications and represent the concepts in different levels of granularity, and mappings under UMLS and OMOP vocabulary may not be fully one-to-one [21]; (2) The biomedical domain is dynamic and evolving and involves the need to periodically adjust semantic meanings [22]. Due to the mechanisms of lexically based and semantically based methods, the final mapping results might not be able to adapt to the change. (3) The quality and completeness of mappings are usually unknown [23]. Therefore, to use the mappings defined in UMLS and OMOP vocabulary in response to clinically derived questions, it is necessary to evaluate the mapping coverage and quality.

For this study, we built and evaluated our terminology mapping using the Medical Dictionary for Drug Regulatory Activities (MedDRA) and the International Classification of Diseases (ICD) as an illustration. The linkage between MedDRA and ICD is essential for drug safety and pharmacovigilance research. MedDRA is a standardized medical terminology developed to capture regulatory information about medicinal products. It is also a recommended terminology for adverse event reporting in several data sources, such as the Federal Drug Administration (FDA) Adverse Event Reporting System (FAERS) [24], Canada Vigilance database, and EudraVigilance database. The FAERS is a voluntary reporting system that is designed to support the FDA’s post-marketing safety surveillance program for drug and therapeutic biologic products [24]. FAERS, however, has certain limitations, such as missing and unverified data, duplicated and incomplete reports, and no established causation between drugs and adverse events [25]. To verify the safety signal detected from FAERS, we need to screen for patients who receive the same medications and compare their adverse reactions with those found in other longitudinal observational databases. Electronic health records/electronic medical records (EHRs/EMRs) data are desired options, as they contain numerous observational medical data from inpatient and outpatient visits. Standard codes, such as ICD and the Systematized Nomenclature of Medicine-Clinical Terms (SNOMED CT), are commonly used to record a patient’s medical condition and intervention.

Considerable research has attempted to associate MedDRA with SNOMED CT [5, 11, 15, 16, 18, 26]. Bodenreider et al. [26, 27] utilized UMLS as a dictionary to study the mapping relationships between MedDRA and SNOMED CT. One study found that 64.6% of MedDRA preferred terms can be mapped to SNOMED CT [11]. Despite the fact that MedDRA-to-SNOMED CT mapping has a high coverage rate, SNOMED CT is used by only 10–30% of EHR vendors at least until the year of 2012 [5]. To enable the retrospective study of EHR data prior to the wide spread of SNOMED CT, ICD is a more ideal candidate as it’s frequently used by healthcare providers and has been incorporated into many EHRs and EMRs as diagnosis codes for decades.

Fewer research, however, has studied mapping between MedDRA and ICD [6, 7, 27]. One study employed UMLS to annotate ICD-9 codes to MedDRA [27]. Another study was the only one that attempted to automatically map ICD to MedDRA based on UMLS [6]. None of these studies, however, has systematically evaluated the quality of the mapping provided by UMLS. Some research also uses the OMOP CDM to transform ICD codes to either SNOMED CT or MedDRA without evaluating the quality [28]. Our study investigates the current status of mapping between MedDRA and ICD in two ways. First, in terms of coverage evaluation, we investigate the mapped terms between the two terminologies based on UMLS and the OMOP vocabulary. Second, we sampled the unmapped terms in UMLS and summarize the unmapped situations to guide future improvement of the mappings. Since 2015, ICD-10-Clinical Modification (ICD-10-CM) has gradually replaced ICD-9-CM as the reimbursement code [19]; however, for a long time, the predominant coding scheme in many EHR/EMR databases was ICD-9-CM [5]. Thus, it is important to study the mapping status between MedDRA and ICD-9-CM/ICD-10-CM for retrospective study.

## Results

### Mapping statistics

The created mapped pairs are MedDRA PT–ICD-9-CM, MedDRA PT–ICD-10-CM, MedDRA LLT–ICD-9-CM, and MedDRA LLT–CD-10-CM. After removing duplicates, there were 4609 MedDRA PT terms and 18,664 MedDRA LLT terms that had a mapping to at least one ICD term in UMLS as well as 4078 unique MedDRA PT terms and 246 LLT terms in OMOP vocabulary.

After combining all UMLS and OMOP mappings, there were 19,860 unique terms. A cross-check with the MedDRA 23.1 release indicated that these included 5726 PT and 19,860 LLT terms. The analysis showed that a total of 6413 unique PT terms were mapped in either UMLS or OMOP, covering 27.23% of all MedDRA PT terms (Table 1).

**Table 1:** Mapping summary from UMLS and OMOP CDM

	MedDRA PT		MedDRA LLT	
Type of mapping	UMLS	OMOP	UMLS	OMOP
MedDRA terms mapped from ICD-9-CM	2788	3308	13,458	200
MedDRA terms mapped from ICD-10-CM	3819	3542	13,851	213
Union of unique MedDRA terms from UMLS and OMOP	5726		19,860	
Union of unique MedDRA PT terms after converting LLT to PT	6413			

*Note.* The values in each column refer to the number of terms

**Fig. 1:**
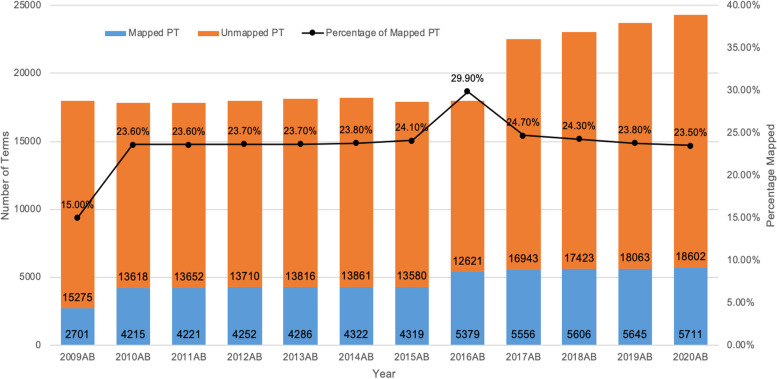
UMLS PT term mapping statistics for 2009–2020

The trend of MedDRA-ICD mappings in UMLS between the years 2009 and 2020 was shown in Fig. 1. With the increase in terms each year in the UMLS Metathesaurus, the percentage of terms that are mapped decreased slowly from 2016 to 2020.

**Fig. 2:**
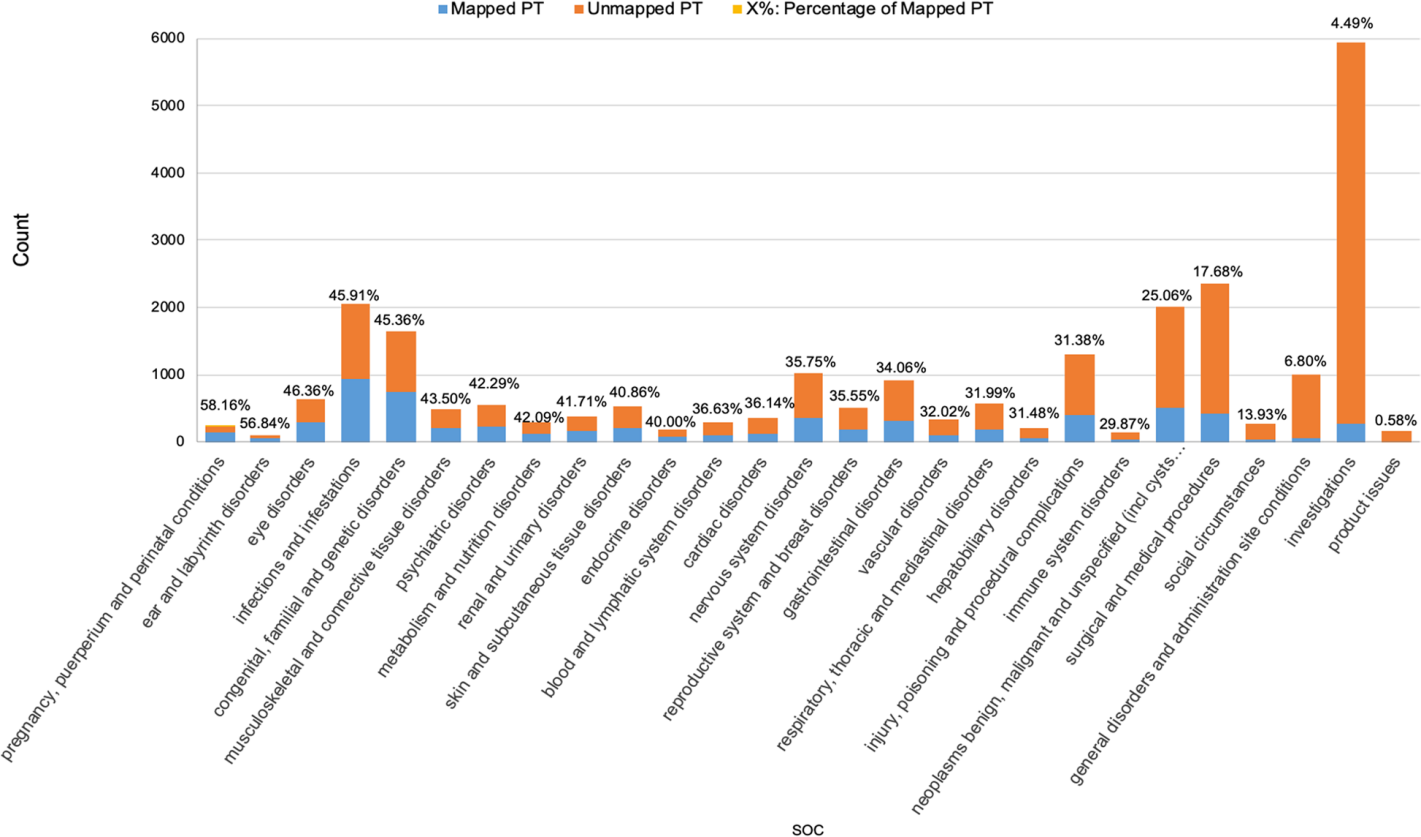
Summary of mapped and unmapped terms under 27 SOCs

As noted, each MedDRA PT belongs to at least one of the 27 SOC categories. We also summarized the mapped and unmapped PT terms and their mapping percentage under each SOC level (Fig. 2). SOCs “Pregnancy, puerperium, and perinatal conditions,” “Ear and labyrinth disorders,” and “Congenital, familial, and genetic disorders” had mapping percentages above 50%, the highest among all 27 SOCs. SOCs “General disorders and administrative site conditions” and “Investigations and product issues” had the lowest mapping coverage, below 10%.

### Evaluation of mapped group

All 1804 mapped pairs were reviewed independently by two annotators, and their Cohen-Kappa inter-rater reliability is 0.803. Following a comparison of ICD and MedDRA terms, 51.44% of the 1804 matched terms in UMLS were found to be an “Exact match”. Another major category is “PT term broader than ICD term,” for which 42.13% of the matched terms were categorized as such. After the first-round independent evaluation, two annotators had a discussion session and reached 100% agreement on all 1804 mapped pairs. The final evaluation summary is shown in Table 2.

**Table 2:** Evaluation Summary of Mapped Terms

Mapping relationship for the mapped group	Number	Percentage
Exact match	928	51.44%
PT term narrower than ICD term	68	3.77%
PT term broader than ICD term	760	42.13%
Partial overlap	48	2.66%
Total	1804	100%

### Evaluation of unmapped group

The results of the evaluation of unmapped pairs are summarized in Table 3. We randomly selected 100 terms under each SOC. The number of PT terms from some SOCs is less than 100; hence, a total of 2400 PT terms were randomly selected for our experiment.

**Table 3:** Evaluation Summary of Mapping Relationships for the Unmapped Group

Mapping relationships for the unmapped group	Number	Percentage
Exact match	56	2.33%
PT term narrower than ICD term	806	33.58%
PT term broader than ICD term	217	9.04%
Partial overlap	378	15.75%
Totally irrelevant	907	37.79%
Other reasons	36	1.50%
Total	2400	100.00%

## Discussion

Since 2010, the number of terms added each year into the UMLS Metathesaurus has been increasing. Before the 2016 release, there were no LLT mappings in UMLS. With this increase, however, the percentage of terms that are being mapped has been slowly decreasing since 2010, which shows the need to develop more mapping relations between terms. At least two versions of the UMLS are being updated every year, our annotation studies sought to use the most updated version available. Hence, different versions of the UMLS Metathesaurus files can be seen in our paper.

The quality evaluation of MedDRA-ICD mapping was focused on the PT level. PT terms are preferred for the majority of adverse event reporting systems. Take FAERS from FDA for example, all the adverse reaction were recorded on the PT level. In OMOP CDM, we only found around 200 mapped pairs between LLT and ICD. Although there are over 3000 mapped pairs of LLT to ICD pairs from UMLS, but according to the nature of MedDRA structure, all PT terms are self-contained at the LLT level. So many of those 3000 pairs are the same as PT terms.

For the MedDRA PT terms under the 27 SOCs, “Investigations” has the highest number of PT terms of all SOCs, but only 4.82% were mapped to ICD terms through either UMLS or OMOP vocabulary. The term “Investigations” describes concepts related to medical conditions and qualitative results. One reason for this high rate of unmapped PT terms is that the terms under this SOC could be out of the scope of ICD. Given that some investigation terms have similar linguistic structures, however, future mapping steps can be specifically tailored to identify the procedure and corresponding results for each term.

There are limitations associated with the use of the UMLS Metathesaurus. Through the process of annotation, the ranking function provided by UMLS has limited performance. For some terms, the best-matched term annotated by our annotator do not appear within the top 1000 returned results by the API. Because the back-end algorithm used by the UMLS API is not open, it’s difficult for reseachers to analyze the mapping relationships. Therefore, we used our self-developed ranking functions for the remaning annotation.

For the annotation process, we sampled only 100 unmapped PT terms under each SOC due to the time and effort required of domain expert annotators. Even though we used random selection, it may not be sufficient to represent the distribution pattern of the mapping relationship for the entire set of PT terms. Further, the annotation can sometimes be subjective, reflecting the mapping category percentage results. For instance, under the “PT term narrower than ICD term” category, often, long stretches of similarly themed terms, such as “methanol, ethanol, isopropanol,” appear consecutively after an overarching term, such as “alcohol.” In such cases, a difference in judgment for “narrower vs. broader” directionality may affect multiple mapped pairs in close proximity, amplifying the effect of inter-rater subjectivity for this category.

### Quality analysis of mapped pairs

Only 51.44% of mapped pairs were rated as an “Exact match,” indicating that our annotators might have more strict standards than those of UMLS. They annotated pairs as an “Exact match” only if the two terms were lexically identical terms, were with a single-word modifier inconsequential to conceptual meaning, or had insignificant variations in word order. For example, “thromboangiitis obliterans” and “Buerger’s disease” reference the same pathophysiological process. “Polyneuropathy idiopathic, progressive” and “idiopathic polyneuropathy, progressive” differ only in word order.

An example of “PT term broader than ICD term” for the mapped group can be found when a broader PT term, e.g., “drug abuse,” is mapped to the ICD term that is referencing narrower subcategories of drug abuse, e.g., “opioid abuse,” “cocaine abuse.” Analogous logic applies to the “PT term narrower than ICD term” category. “Partial overlap” applies to terms such as “pilonidal cyst” versus “pilonidal abscess,” for which neither term inherently falls within the range of another, but both reference relevant concepts, e.g., similar disease states, similar organ systems.

Among the non-exact matches, the “PT term broader than ICD term” category yielded the most results, suggesting that, on average, ICD terms were narrower than were MedDRA terms on the PT level. Clinically, this alludes to the ICD system’s utility in charting and diagnosing more specific disease processes. Another reason for the disagreement could be annotator bias. Subjectivity may occur during the annotation process, such as specialty-specific preferences, training biases, and so on.

Nevertheless, the 0.803 Cohen-kappa score indicates high inter-rater agreement. Following an independent rating stage, the annotators determined the reasons for each disagreement term by term (e.g., misinterpretation of pathophysiology, initial misreading of term) and made corrections where appropriate.

### Possible improvement for the unmapped group

For the evaluation of unmapped terms, the different granularities of the two coding systems could explain the causes for “PT term broader than ICD term” and “PT term narrower than ICD term,” e.g., “Thyroid diseases” from ICD and “Haemorrhagic thyroid cyst” from PT terms. There are, however, 56 unmapped terms identified by our evaluators as an “Exact match” out of the 2400 sampled PT terms. These 56 “Exact match” pairs indicate room for improvement of the UMLS mapping.

Most of the “Partial overlap” relationships appear when the PT and ICD codes use different expressions for a similar disease. For instance, for the PT term “Liver and pancreas transplant rejection” under the “Immune system disorders” SOC category, the best ICD-9-CM match that the annotator provided is “Complication of transplanted pancreas.” We place these types of cases in the “Partial overlap” category. It should be noted that the mapping relationships are not always one to one. More than two ICD codes could be the best match for one PT term.

“Totally irrelevant” relationships occur often for certain SOCs. For “Product issues,” “Social circumstances,” and “Investigation” SOCs, for example, almost all of their PT terms were classified under the “Totally irrelevant” relationship. This reflects the difference in granularity and focus between MedDRA and ICD. Patterns also appear for other relationship categories. The top-matched ICD codes for the PT terms under the “Neoplasms benign, malignant, and unspecified (including cysts and polyps)” SOC are usually in the outer range of PT terms. One possible reason is that ICD usually uses a broad definition for cancer diseases.

It is also worth noting that, as seen in Tables 2 and 3, the relative results of narrow-to-broad and broad-to-narrow mappings are different among the unmapped and mapped groups. This difference does not confict with our conclusion that ICD terms are, in general, narrower than MedDRA terms on the PT level. The ranking algorithm would find the best-matched term based on the current string instead of adding details, such as body position, that ICD codes usually have. This will result in best-matched ICD codes’ being broader than PT terms, which is why the percentage of “PT term narrower than ICD term” is much higher.

## Conclusion

The overall percentage of PT terms mapped through either UMLS or OMOP vocabulary is 27.23% of all MedDRA PT terms. We evaluated the mapped pairs through the CUI in UMLS and determined that only 51.44% are considered as “Exact match” by our annotators. We further evaluated the 2400 sampled unmapped terms and determined that 56 of the PT terms have “Exact match” pairs, suggesting the expansion capacity for MedDRA to ICD mapping. The same mapping relationships identified in both mapped and unmapped groups in UMLS, “PT term narrower than ICD term,” “PT term broader than ICD term,” and “Partial overlap,” suggest that the use of UMLS as a mapping guideline may require further examination of the “Exact match” relationship. Some of the mapped pairs found in UMLS between MedDRA and ICD are not strictly “Exact match” due to differences in granularity and focus. For 72% of the unmapped PT terms, the identified “Exact match” pairs illustrate the possibility of identifying more mapping pairs. Referring to UMLS’s own mapping standard, some of the 44.95% “broader” and “narrower” relationships we identified in unmapped terms should qualify for the expansion of MedDRA to ICD mapping. The overall “Exact Match” pairs we have identified can serve as a dictionary for the researcher trying to identify the adverse reaction from billing codes. The entire process of retrieving and evaluating terminology mappings can also be applied to other scenarios.

## Method

### Terminology mapping

To summarize the current mapping status of MedDRA to ICD, we calculated the mapping statistics from UMLS and OMOP vocabulary (UMLS Metathesaurus 2020AB and OMOP CDM v5). We used the relationship table from OMOP standardized vocabularies to map two terminologies. Almost all of the MedDRA terms in the UMLS and OMOP are at the preferred term (PT) and lowest-level term (LLT) levels (PT terms are the parent nodes of LLT terms). We cross-referenced the mapping from UMLS and OMOP vocabulary with the official MedDRA data, searching by string. Because ICD-9-CM and ICD-10-CM have both been used in EHR/EMR systems, we created multiple mapped pairs: MedDRA PT–ICD-9-CM, MedDRA PT–ICD-10-CM, MedDRA LLT–ICD-9-CM, and MedDRA LLT–ICD-10-CM.

To calculate the mapping coverage, once the mapped pairs were extracted, we converted all the LLT terms in the mapped pairs to the PT level, referring to M﻿edDRA Distribution File Format Document Version 23.1. In MedDRA, each LLT term is under a PT term, and all PT terms are self-contained at the LLT level. Using this property, we calculated the overall percentage of mapping coverage of MedDRA-ICD pairs on the PT level. The number of uniquely mapped terms and their percentage on both PT and LLT levels were calculated for UMLS and OMOP vocabulary. The detailed description of our mapped pairs retrieval was provided in the appendices.

**Fig. 3:**
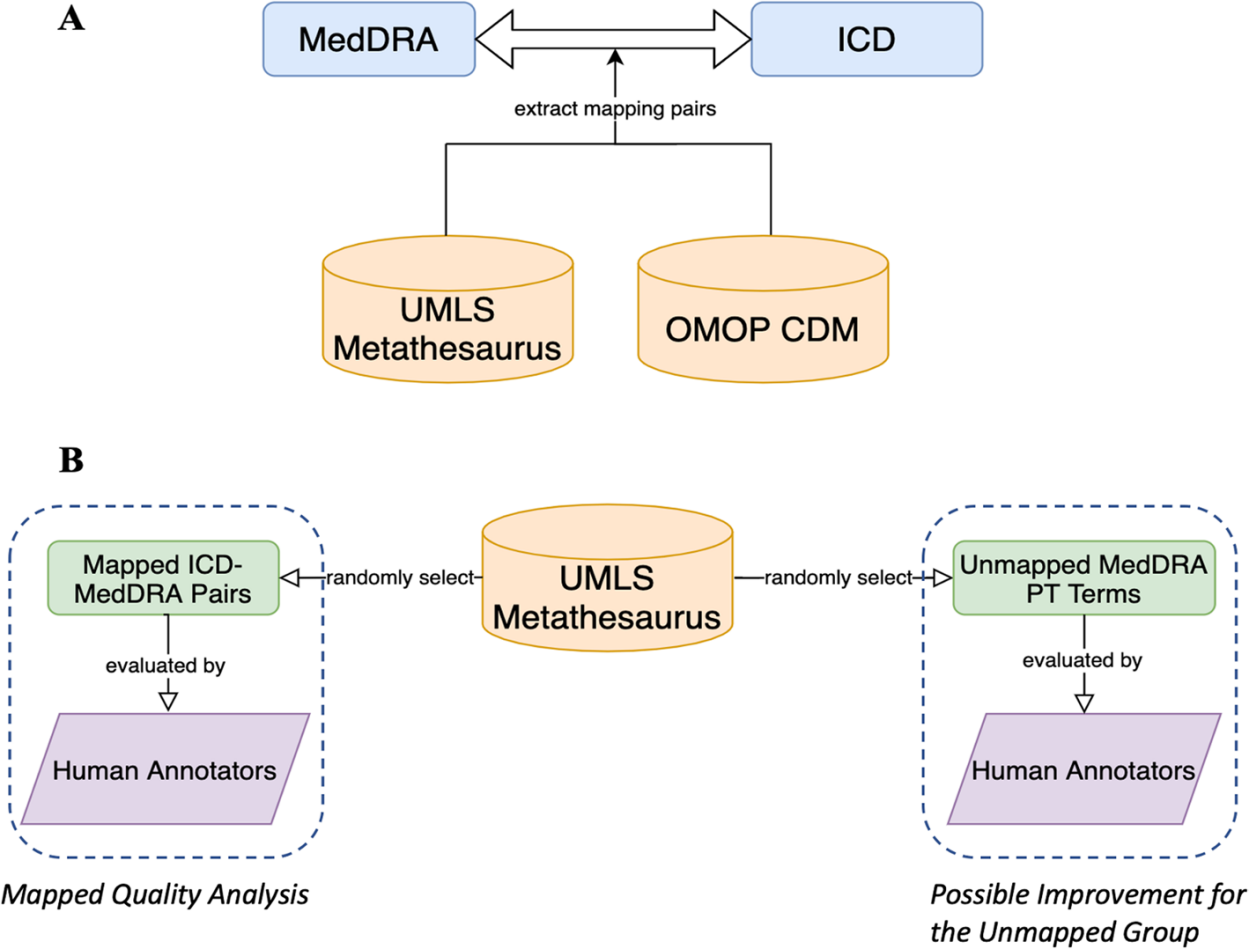
Terminology mapping and evaluation framework. **A**: Coverage evaluation using UMLS and OMOP Vocabulary; **B**: Further quality evaluation for UMLS mappings

Then, we conducted a quality evaluation of the mapping coverage by randomly selecting 10% of the mapped PT terms and 100 unmapped PT terms under each system organ class (SOC). The mapped pairs were extracted from UMLS. These PT terms came before the aggregation of LLT terms, which means all the LLT-ICD mappings were excluded. Four annotators (JD, AG, DT, and HE) with a clinical background were divided into two teams to find the mapping relationship/unmapped reasons for each selected PT term. Figure 3 shows the framework of the terminology mapping and evaluation process.

### Mapped pairs retrieval

Our method of extracting direct mappings is based on the CUI from MR1CONSO.RRF. Although multiple mapping files could be found in UMLS, some files still need further investigation and clarification. Many concepts linked by the relationship “source asserted synonymy” (REL = SY) and “related and possibly synonymous” (REL = RQ) from MRREL.RRF and MRMAP.RRF have the same CUI. In the 2020AA release, 37.56% of relationships with RQ and 99.14% with SY have the same CUI. For the relationships with RQ that do not have the same CUI, 81.65% have the RELA of “classifies/classified as,” while only 2.47% have the RELA of “mapped to/mapped from.” Therefore, we used only the CUI as the mapping criteria.

We extracted the MedDRA-ICD mapped pairs, using source abbreviations of ICD-9-CM, ICD-10-CM, and MedDRA for all of the CUIs from MRCONSO.RRF. As described in the official documentation [29], most synonymous concepts in MRREL.RRF for which REL = RL are self-referential when they have the same CUI. No direct mapping was found between ICD-9-CM/ICD-10CM and MedDRA in the 2020 release of MRMAP.RRF. We also calculated the trend of MedDRA-ICD mappings in UMLS between the years 2009 and 2020.

In addition to direct mapping, we used indirect mapping for OMOP vocabulary. The standard vocabularies from OMOP vocabulary store all terminologies in the CONCEPT table, and semantic relationships between terms are defined in the CONCEPT_RELATIONSHIP table. To extract mapped pairs in OMOP vocabulary, in addition to direct mappings, we include indirect mappings. In the indirect mapping method, MedDRA is first mapped to SNOMED, then to ICD, using “MedDRA–SNOMED eq” and “Maps to” concept relationships. We used predefined “MedDRA–ICD” mapping relationships in the CONCEPT_RELATIONSHIP table for the direct mapping and integrated the results with indirect mapped pairs.

### Candidate-searching algorithm for unmapped terms

To identify additional mapped pairs, we developed a ranking algorithm to recommend the best ICD mapping candidates for the PT term. We first extracted 40,855 entries under the source name “ICD9CM” from the “MRCONSO.RRF” file of UMLS 2018AA. Then we formatted the entries and their synonyms with the same CUI into a dictionary and ranked the ICD codes for each PT term (i.e., query), using the following information retrieval-based methods [30–33]: (1) invoke Lucene APIs to index all of the PT terms and their synonyms with concept IDs; and (2) employ the BM25 model [34] provided by Lucene to retrieve the top 25 candidate ICD codes from the index.

### Evaluation

**Fig. 4:**
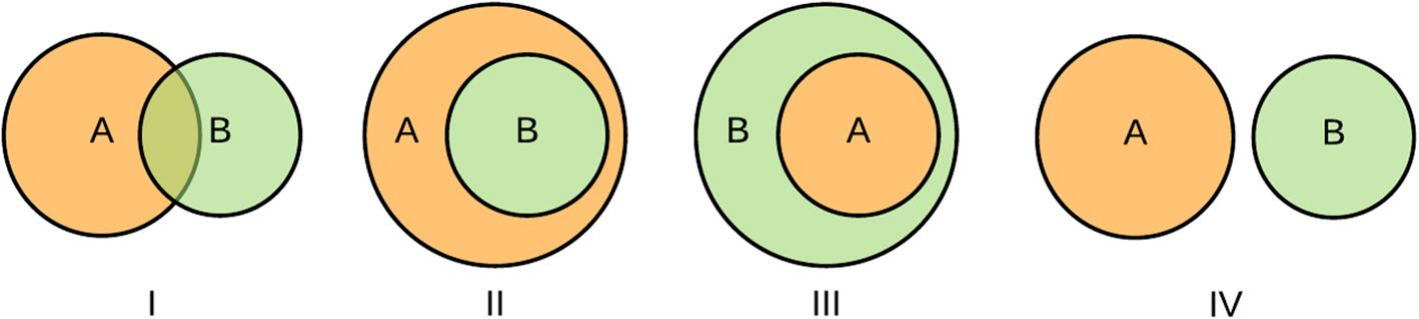
Relationships of semantic scope between two non-synonymous concepts

To assess the precision and recall of the current mapping status, we created the evaluation criteria based on a Venn diagram, which shows all possible logical relations between a finite collection of different sets [35]. Fig. 4 shows the relationships of the semantic scope between two non-synonymous concepts (i.e., A and B), using a Venn diagram: A partially overlaps with B (I), A is broader than B (II), A is narrower than B (III), and A is irrelevant to B (IV). In addition to these four types of relationships, we identify another situation in MedDRA-ICD mapping in which A equals B. We used these defined relationships to evaluate the mapping status of two groups: mapped and unmapped. Among these groups, 10% of the mapped MedDRA PT-ICD pairs from UMLS 2019AA were randomly selected as the mapped group, and 100 randomly chosen PT terms under each SOC category that are not mapped by UMLS constituted the unmapped group.

We recruited two annotators to investigate MedDRA-ICD pairs from the mapped group. The mapped pairs were sub-classified into “Exact match,” “PT term narrower than ICD term,” “PT term broader than ICD,” and “Partial overlap” categories. They must choose which category each mapped pair belongs to.

To evaluate the mapping with respect to the recall measure, we sampled terms from the unmapped group. We recruited another two experienced clinicians to classify these terms into seven categories accordingly: “Exact match,” whereby the following situations are included: lexically identical terms, terms that difer by a single-word modifier inconsequential to conceptual meaning, or insignificant variations in word order; “PT term broader than ICD term,” “PT term narrower than ICD term,” “Partial overlap,” “Totally irrelevant,” “No response,” or “Other reasons,” where “No response” means that there were no returned results from searching the UMLS Metathesaurus. We utilized the “approximate match” function from the UMLS Metathesaurus and our self-developed computer algorithm to compare the unmapped terms from MedDRA and similar terms from ICD codes, as described previously.

To ensure the quality of manual annotation, for both evaluations, we first assigned reviewers the same 100 PT terms to annotate, and calculated the inter-rater reliability score between them. We proceeded after 80% agreement had been achieved.

## Appendices

### Data and materials

#### MedDRA

MedDRA has a five-level hierarchical structure. The top level is the SOC, representing 27 broad classes grouped by etiology, manifestation site, and purpose [36]. The most frequently used level of terms is PTs, which are distinct and unambiguous descriptors. Clinical pathologic or etiologic qualifiers are represented in this level. The lowest level is LLTs, with maximum specificity. In MedDRA, PT terms are the parent nodes of LLT terms, and every PT has one identical LLT for data entry purposes. In addition, LLTs may be synonyms, lexical variants, quasi-synonyms, or sub-elements of their PTs.

#### ICD

The ICD is a medical classification system for diseases, laboratory findings, and causes of injury and disease. ICD-CM is the United States’ clinical modification of the ICD codes [37]. ICD-9-CM is used to code and classify morbidity data from inpatient and outpatient records [38]. It contains a classification system for surgical, diagnostic, and therapeutic procedures [39].

**Fig. 5:**
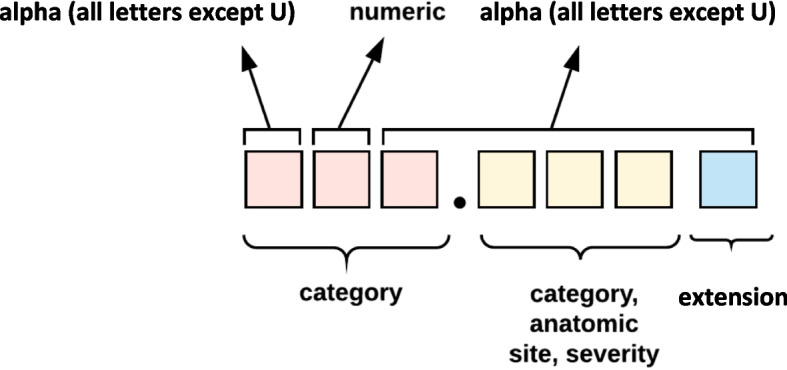
Composition of ICD-10-CM code

ICD-10-CM replaced ICD-9-CM to include more health conditions. As ICD-10-CM’s main component, the tabular list presents codes categorized into 21 chapters based on body system or condition [40]. Figure 5 shows the composition of the 7-character code of ICD-10-CM.

#### UMLS Metathesaurus

The UMLS Metathesaurus is a common vocabulary that links disparate biomedical terminologies. It utilizes a CUI to link synonymous terms and identifies useful relationships between concepts and preserves the meanings, concept names, and relationships from each vocabulary set [41]. These relationships are recorded in multiple files, such as MRCONSO.RRF, MRREL.RRF, and MRMAP.RRF. MRCONSO.RRF contains information about each unique concept name in the Metathesaurus, whereby each term is assigned a CUI. Asymmetrical relationships are specified in MRREL.RRF, and pair-wise mappings are in MRMAP.RRF (simpler mappings are in MRSMAP.RRF). Relationships and mappings in MRREL.RRF and MRMAP.RRF are described by using the attribute relationship (REL) and relationship attribute (RELA), a more specific description of a given relationship.

We summarized the mapping statistics of the UMLS Metathesaurus from 2009 to 2020. If UMLS released two versions of a distribution file in a year (marked as “AA” and “AB”), we used both versions, subject to the latest version at the time of our research. Within UMLS, mapped terms from MedDRA to ICD are found using the CUI, which links terms with the same meaning [11].

#### Observational medical outcomes partnership common data model (OMOP CDM)

Observational Health Data Sciences and Informatics (OHDSI) developed OMOP CDM to better assist researchers who use observational data for post-marketing drug safety surveillance [5]. The CDM can integrate disparate data sources and further classifies medical vocabularies from different sources into one common format [7]. It also provides a relationship table that encodes pre-identified mapped pairs across various terminologies. We extracted the mapping pairs from the OMOP vocabulary using SNOMED as an intermediate terminology.

## Data Availability

The data that support the findings of this study are available from UMLS’s (https://www.nlm.nih.gov/research/umls/licensedcontent/umlsknowledgesources.html) and OHDSI’s website (https://www.ohdsi.org/data-standardization/the-common-data-model/) but restrictions apply to the availability of these data, which were used under license for the current study.

## Abbreviations

FAERS: Federal Drug Administration Adverse Event Reporting System
ICD: International Classification of Diseases
LLT: Lowest-level Term
MedDRA: Medical Dictionary for Regulatory Activities
OHDSI: Observational Health Data Sciences and Informatics
OMOP CDM: *Observational Medical Outcomes Partnership Common Data Model*
PT: Preferred Term
SOC: System Organ Class
SNOMED-CT: Systematized Nomenclature of Medicine-Clinical Terms
